# Sustained Shugoshin 1 downregulation reduces tumor growth and metastasis in a mouse xenograft tumor model of triple-negative breast cancer

**DOI:** 10.1186/s13008-023-00088-5

**Published:** 2023-04-30

**Authors:** Shirley Jusino, Yainyrette Rivera-Rivera, Camille Chardón-Colón, Patricia C. Rodríguez-Rodríguez, Janeishly Román-González, Valeria S. Juliá-Hernández, Angel Isidro, Qianxing Mo, Harold I. Saavedra

**Affiliations:** 1 Department of Basic Sciences, Ponce Health Sciences University-Ponce Research Institute, 395 Zona Industrial Reparada 2, Ponce, Puerto Rico 00716-2348 USA; 2 Department of Biology, University of Puerto Rico-Ponce, 2151 Avenida Santiago de los Caballeros, Ponce, Puerto Rico 00716 USA; 3 Department of Basic Sciences, Division of Physiology, Ponce Health Sciences University-Ponce Research Institute, 395 Zona Industrial Reparada 2, Ponce, Puerto Rico 00716-2348 USA; 4 Department of Biostatistics and Bioinformatics, H. Lee Moffitt Cancer Center, 12902 USF Magnolia Drive, Tampa, Florida 33612 USA; 5 Department of Basic Sciences, Division of Pharmacology and Cancer Biology, Ponce Health Sciences University-Ponce Research Institute, 7004, Ponce, Puerto Rico 00732-7004 USA

**Keywords:** SGO1, Tumor growth, Metastasis, Epithelial-to-mesenchymal transition, Triple-negative breast cancer, Mitosis

## Abstract

**Background:**

Triple-negative breast cancer (TBNC) is an aggressive breast cancer subtype with a poor prognosis. Shugoshin-1 (SGO1) protects chromatids from early separation. Previous studies from our group have demonstrated that transient SGO1 downregulation suppresses early stages of metastasis (the epithelial-to-mesenchymal transition, or EMT, cell invasion, and cell migration) in TNBC cells. Thus, the inhibition of SGO1 activity may represent a potential therapeutic intervention against cancers that progress to metastasis. Therefore, we aimed to investigate the effects of sustained shRNA-mediated *SGO1* downregulation on tumor growth and metastasis in TBNC. To that end, female NOD-SCID Gamma (NSG) mice were injected with 2.5 × 10^6^ shRNA Control (n = 10) or shRNA *SGO1* (n = 10) MDA-MB-231 cells. After eight weeks, the number of mice with metastasis to the lymph nodes was calculated. Primary and metastatic tumors, as well as lung and liver tissue, were harvested, measured, sectioned, and stained with hematoxylin and eosin (H&E) stain.

**Results:**

Tumor growth and metastasis to the lymph nodes and lungs were significantly reduced in the shRNA *SGO1*-treated mice group, while metastasis to the liver tends to be lower in cells with downregulated SGO1, but it did not reach statistical significance. Furthermore, sustained SGO1 downregulation significantly reduced cell proliferation, cell migration, and invasion which correlated with lower levels of *Snail*, *Slug*, *MMP2*, *MMP3*, and *MMP9*.

**Conclusion:**

The supression of SGO1 activity in TNBC harboring dysregulated expression of SGO1 may be a potential target for preventing breast cancer growth and metastasis.

**Supplementary Information:**

The online version contains supplementary material available at 10.1186/s13008-023-00088-5.

## Background

Breast cancer is the most common cancer diagnosed and the number one cause of cancer-related deaths in women worldwide [1]]. Breast cancer can be classified into molecular or pathological subtypes, based on the receptor status of estrogen (ER), progesterone (PR), or Human Epidermal Growth Factor 2 (HER2) [2, 3]. The principal subtypes include HER2 + (ER−/PR− and amplified for Her2), luminal A (ER + /PR + and HER2−), luminal B (ER + /PR + /HER2 + or HER2−), and triple-negative breast cancer (TNBC, ER−/PR−/HER2−).

Our laboratory focuses on the study of TNBC. This subtype represents 15% to 20% of all primary breast cancers and occurs most frequently in African American and Hispanic women at a younger age (< 50 years of age) [4]. Higher frequencies of this subtype, along with the detection of larger breast cancers of higher stages, contribute to poorer survival outcomes in African American and Hispanic women with breast cancers relative to non-Hispanic white women [5]. TNBC is the most aggressive subtype with a higher likelihood to metastasize [6, 7] and women with TNBC have the poorest prognosis when compared to other subtypes with an estimated overall survival of 12–18 months [8]. TNBC remains a clinical challenge despite some improvement in targeted therapy developments that includes immunotherapy, PARP and AKT pathway inhibitors, antibody–drug conjugates, and androgen receptor blockade [4].

A potential therapeutic target for TNBC is Shugoshin-1 (SGO1) or mitotic kinases that interact with SGO1, including Nek2A [9], Bub1 [10], TTK (also known as Mps1) [11, 12], and Aurora Kinase B [13, 14]. Many of these kinases are currently in clinical trials, and early results for TTK inhibitors are promising [15, 16]. The physiological role of SGO1 is to ensure chromosomal stability by protecting the centromeric cohesion of sister chromatids, assisting bi-orientation attachment at the kinetochores, and safeguarding the centriole cohesion engagement during mitosis and meiosis [17]. Optimal levels of SGO1 are needed for proper cell function. On the other hand, the role of SGO1 in cancer is complex and context-dependent. For example, SGO1 expression is decreased in colorectal cancer and SGO1 downregulation causes G2/M arrest, apoptosis, and chromosome instability leading to tumorigenesis [18, 19]. Meanwhile, overexpression of SGO1 is a poor prognostic factor in prostate cancer [20], and hepatocellular carcinoma [21], and its overexpression induces proliferation and metastasis through the AKT signaling pathway [22]. Previous studies from our laboratory showed that SGO1 is overexpressed in breast cancers (including TNBC) and that it correlates with overexpression of the E2F3 transcription factor, and with poor prognostic factors, including centrosome amplification, chromosome instability, and the epithelial-to-mesenchymal transition (EMT) [23–25]. Further, we demonstrated that transient, siRNA-mediated silencing of SGO1 modulates the protein levels and localization of EMT-related proteins, as well as decreases matrix metalloprotease (MMP) 3 mRNA levels. These EMT-associated changes led to reduced cell invasion and migration in TNBC cells MDA-MB-231 and Hs578t, and are dependent on MMP3 and SNAIL [26].

Here, we investigate the effects of SGO1 downregulation in tumorigenesis and metastasis in NOD-SCID gamma (NSG) mice. We explore the effects of downregulation by assessing cell proliferation, viability, apoptosis, and EMT in vitro*.* We hypothesize that SGO1 downregulation would decrease tumorigenesis and metastasis by modulating EMT-related proteins and reducing cell viability.

To test our hypothesis, MDA-MB-231 cells expressing either shRNA Control or shRNA *SGO1* were injected in NSG mice. Our results showed that SGO1 downregulation significantly reduced tumor growth and metastasis of TNBC to the lungs and lymph nodes. In vitro studies using the same cell lines showed that EMT protein expression and cell invasion were associated with the reduction in the levels of EMT transcription factors and MMPs. These results suggest that SGO1 is a potential therapeutic target for TNBC.

## Materials and methods

### Cell culture

The MDA-MB-231 (HTB-26) cell line was purchased from American Type Culture Collection (ATCC, Manassas, Virginia, USA) and transduced with shRNA *SGO1* (sc-106548-V, Santa Cruz Biotechnology, Dallas, Texas, USA) or negative scrambled shRNA Control (sc-108080, Santa Cruz Biotechnology) lentiviral particles. The cells were cultured as described in our previous publications [23, 25, 27].

### Western blotting

To investigate the effects of SGO1 downregulation on EMT protein expression, MDA-MB-231 cells expressing shRNA control or an shRNA targeting *SGO1* were lysed and Western blot protocol was performed according to our published protocols [23]. The following were used as primary antibodies: SGO1 (sc-393993, Santa Cruz Biotechnology), E-cadherin (3195s, Cell Signaling Technology, Danvers, Massachusetts, USA), Slug (9585s, Cell Signaling Technology), Snail (3879s, Cell Signaling Technology), Twist1 (46702s, Cell Signaling Technology), Vimentin (5741s, Cell Signaling Technology), Zeb1 (3396, Cell Signaling Technology), and ZO-1 (8193s, Cell Signaling Technology). β-actin (sc-47778, Santa Cruz Biotechnology) was used as a loading control. Either goat anti-rabbit HRP (sc-2004, Santa Cruz Biotechnology) or goat anti-mouse HRP (NXA931, GE Healthcare, Chicago, Illinois, USA) were used as secondary antibodies. The average of three independent experiments was reported. For original films refer to supplementary material (Additional file 1: Figures S2–S7).

### RNA isolation, RNA quantification, and quantitative real-time PCR

To investigate the effects of SGO1 downregulation on EMT mRNA expression, total RNA was extracted using the RNeasy Mini kit (1002137, Qiagen, Germantown, Maryland, USA) following the manufacturer’s instructions. RNA was quantified and reverse transcribed, and quantitative real-time PCR (qPCR) analysis was performed according to our published protocols [26]. The mRNA levels of *SGO1* (PPH10976A-200, Qiagen), *SNAI1* (Snail, PPH02459B-200, Qiagen), *SNAI2* (PPH02475A-200, Qiagen), *TWIST1* (Twist1, PPH02132A-200, Qiagen), *ZEB1* (Zeb1, PPH01922A-200, Qiagen), *MMP2* (PPH00151B-200, Qiagen), *MMP3* (PPH00235F-200, Qiagen), *MMP9* (PPH00152E-200, Qiagen), *CDH1* (E-cadherin, PPH00135F-200, Qiagen), CDH2 (N-cadherin, PPH00636F-200, Qiagen), VIM (Vimentin, PPH00417F-200, Qiagen), and *TJP1* (ZO-1, PPH09919F-200, Qiagen) were evaluated. *GAPDH* (PPH72843A-200, Qiagen) mRNA levels were used as a control. The 2^−ΔΔCT^ method was used to calculate fold changes relative to the control and the average of three independent experiments was reported.

### In vitro* invasion and migration assays*

BioCoat Matrigel Invasion Chambers (354480, Corning, Glendale, Arizona, USA) and BioCoat Control Cell Culture Inserts (354578, Corning) were used to evaluate the invasion and migration of MDA-MB-231 cells treated with shRNA Control or shRNA *SGO1* using our published methods [28]. Images were taken using the Nikon DS-Ri2 microscope and the average from three independent experiments (four fields/treatments) was reported.

### Viability assay

To investigate the effects of SGO1 downregulation on cell proliferation and viability, a Cell Counting Kit-8 (CCK8) analysis (CK04-11, Dojindo Molecular Technologies, Inc., Rockville, Maryland, USA) was performed as described previously [26]. The average adjusted absorbance from three independent experiments was reported.

### Apoptosis assay

To investigate the effects of SGO1 downregulation in apoptosis, MDA-MB-231 treated with shRNA Control or shRNA *SGO1* were seeded at a density of 1 × 10^6^ and further trypsinized and washed with PBS 1 × before proceeding with the Annexin V Apoptosis Detection Kit I (559763, BD Biosciences, San José, California, USA) protocol. Both cell lines, expressing shRNA Control, or shRNA *SGO1* were prepared as follows for flow cytometry: Unstained, PE Annexin V (no 7-AAD), and 7-AAD (no PE Annexin V). Flow cytometry analysis was performed using the FACSMelody instrument within 1 h of staining. The average from three independent experiments was reported for the analysis.

### Animal model

NSG females (4 weeks old) were obtained from Jackson Laboratories, Inc. (005557). Mice were allowed to adapt under a sterile and pathogen-free environment for two weeks. The mice had free access to distilled water and food were on a 12 h light–dark cycle, and were housed in groups of five per cage. The mice were observed daily by staff, including a veterinarian. For any surgical procedure we used inhaled anesthesia to minimize pain and discomfort. After surgery, the animals were allowed to recover. The parameters to determine a premature endpoint included failure to feed, mood, or groom, or when the tumor reached 10% of body weight.

### Breast cancer xenograft model

To investigate the effects of SGO1 downregulation in tumor growth, a total of 2.5 × 10^6^ MDA-MB-231 (shRNA Control or shRNA *SGO1*) cells were injected into both posterior mammary fat pads of 16–18 weeks old female NSG mice according to our published protocols [26]. To minimize the pain and discomfort, all the procedures were conducted under anesthesia and delivered by mask (2% isoflurane and oxygen). Tumors were measured twice a week for a maximum period of eight weeks (or until mice exhibited severe discomfort). Tumor volume was calculated using the following formula $V=\frac{L \times {W}^{2 }}{2}$, where L represents the larger diameter and W represents the smaller diameter. Mice were euthanized by inhalation of CO_2_ after 8 weeks post-injection or when the mice exhibited severe discomfort and pain evidenced by failure to move, groom, or feed.

### H&E staining

To investigate the effects of SGO1 downregulation in metastasis, the liver, lungs, primary tumors, and secondary tumors were fixed in 10% formalin buffer and embedded in paraffin, as previously described [29]. The tissues were sectioned (2 μm) and stained with hematoxylin and eosin (H&E, Thermo Fisher, Waltham, MA). Images were taken using the Nikon DS-Ri2 microscope and a certified pathologist, Dr. A. Isidro, examined the stained tissues to confirm the presence of micrometastasis in lung and liver tissue, and to evaluate the mitotic index in primary tumors.

### Statistical analysis

For all data analysis, we used the software GraphPad Prism version 8.4.3 (686). The values are represented as mean + /− SEM for n = 3 for all in vitro experiments and n = 10 per group for in vivo experiments. The data were considered significant if the p-value was < 0.05. A Student t-test (unpaired, two-tailed) was used to compare differences between groups. A two-way ANOVA was used to compare differences in groups with more than one categorical variable. For the metastasis studies, a Fisher exact test was performed. To determine the appropriate sample size for measuring tumor growth, we performed the following power analysis: a sample size of 8 mice per group will have 95% power to detect a mean difference of at least 0.3 (with SD = 0.1) using the Hsu (With Best) multiple comparison test at a two-sided 5% type I error using one-way ANOVA.

## Results

### SGO1 downregulation reduces tumor growth and metastasis in NSG mice

**Fig. 1:**
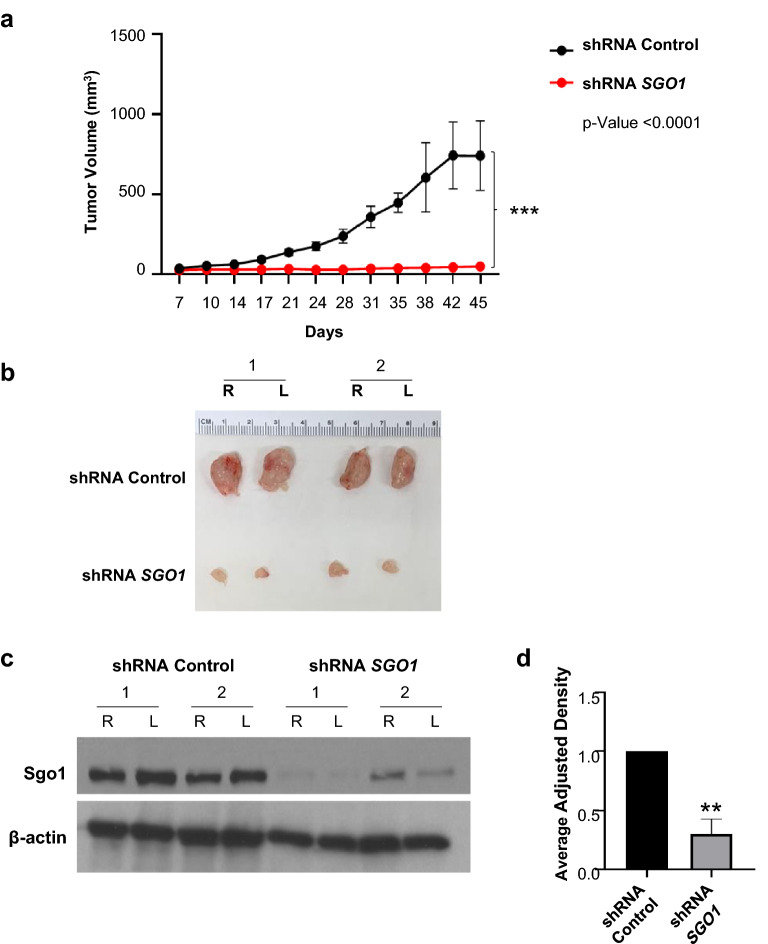
SGO1 downregulation reduces tumor growth. The average of left and right tumors per day for the shRNA Control group (n = 10) and the shRNA *SGO1* group (n = 10) were recorded for 45 days post-injection (**a**). The error bars represent the mean ± SEM. *p < 0.05, **p < 0.01, and ***p < 0.001 were considered statistically significant. A two-way ANOVA was conducted to compare differences between groups with more than one categorical variable. Some representative images of the primary tumors per each group are shown (**b**). A Western blot was performed to characterize the SGO1 expression in representative primary tumors (**c**). The densitometry analysis is shown (**d**). The error bars represent the mean ± SEM. *p < 0.05, **p < 0.01, and ***p < 0.001 were considered statistically significant. N.S. stands for not significant. A Student t-test (unpaired, two-tailed) was used to compare differences between groups

Previous studies from our laboratory have shown a role for SGO1 in early metastasis (the epithelial-to-mesenchymal transition, or EMT, cell migration, and invasion) of TNBC cell lines [25]. However, it is unknown if the stable downregulation of SGO1 would suppress tumor growth and metastasis of TNBC. The results from Fig. 1a show that SGO1 downregulation abrogates tumor growth of MDA-MB-231 cells when compared to shRNA control-treated cells. A representative picture of the tumors is shown in Fig. 1b, while images from other tumors can be found in supplementary materials (Additional file 1: Figure S1a, b). Moreover, the stable knockdown of SGO1 is maintained even after 45 days post-injection (Fig. 1c, d).

**Fig. 2:**
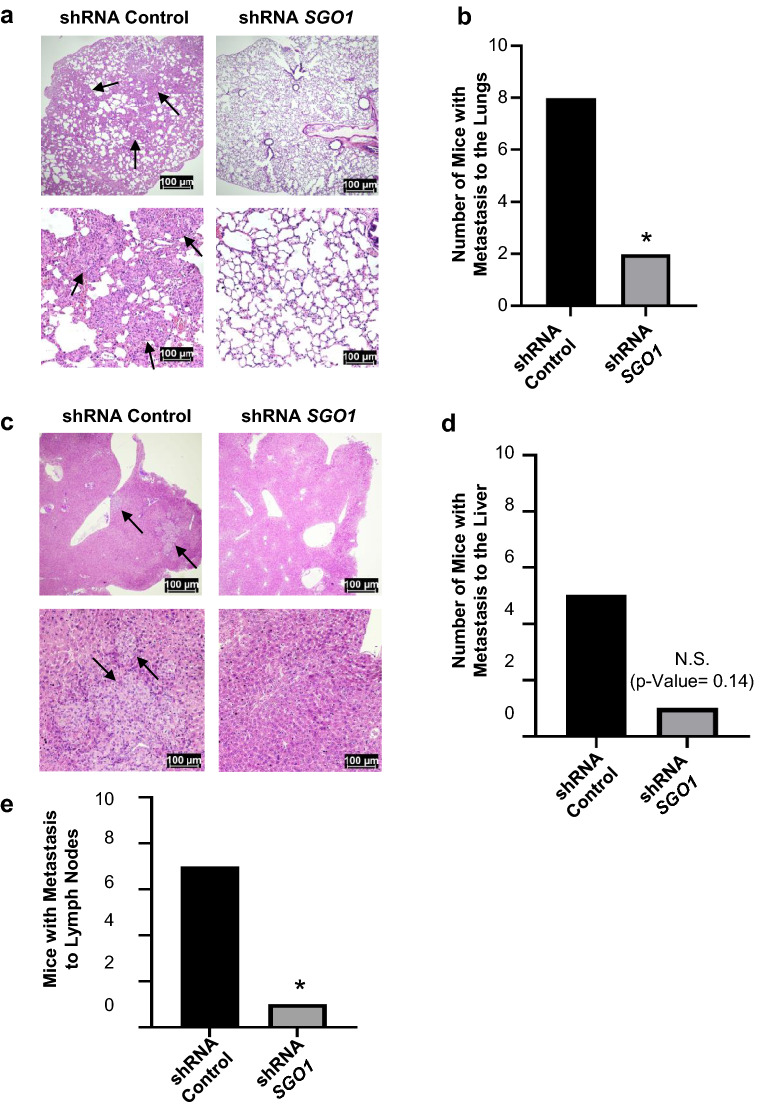
SGO1 downregulation reduces metastasis. Representative images for H&E staining of lung tissue are shown in low (top) and high (bottom) magnification **(a)**. The black arrows point to metastasis (scale bars = 100 μm). The graph shows the number of mice that developed metastasis to the lungs (**b**). Representative images for H&E staining of liver tissue are shown in low (top) and high (bottom) magnification (**c**). The black arrows point to metastasis (scale bars = 100 μm). The graph shows the number of mice that developed metastasis to the liver (**d**). Graph showing tumor volume of secondary tumors (**e**). *p < 0.05, **p < 0.01, and ***p < 0.001 were considered statistically significant, while N.S. is non-significant. A Fisher Exact Test was used to compare differences between groups

Metastasis to the lungs, livers, and lymph nodes was then detected. Metastasis to the lungs is more likely to occur in the shRNA control group, compared to the shRNA SGO1 group (metastasis rate 0.8 vs. 0.2, p = 0.023), Figs. 2a, b. Metastasis to the liver tends to be more likely to occur in the shRNA control group, compared to the shRNA *SGO1* group although it does not reach statistical significance (metastasis rate: 0.5 vs. 0.1, p = 0.14), Figs. 2c, d. Metastasis to lymph nodes is more likely to occur in the shRNA control group, compared to the shRNA *SGO1* group (metastasis rate: 0.7 vs. 0.1, p = 0.02), Fig. 2e. To summarize, these results suggest an important role for SGO1 in tumor growth and metastasis in TNBC.

**Fig. 3:**
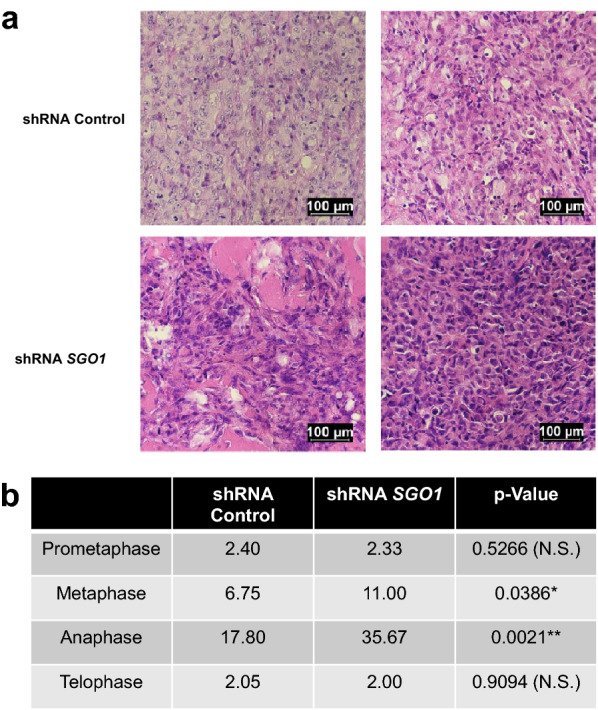
SGO1 depletion increases the number of cells arrested in metaphase and anaphase within tumors. Representative images of primary tumors of NSG mice treated with MDA-MB-231 shRNA Control or shRNA *SGO1* (**a**). Images were taken at 400X magnification (scale bars = 100 μm). A summary table shows the average of cells in each mitotic phase and p-Values (**b**). *p < 0.05, **p < 0.01, and ***p < 0.001 were considered statistically significant. A Student t-test (unpaired, two-tailed) was used to compare differences between groups. *p < 0.05, **p < 0.01, and ***p < 0.001 were considered statistically significant. N.S. stands for not significant

Furthermore, we investigated the effects of shRNA *SGO1* in primary tumors and found significant differences in cells on metaphase and anaphase but not on prometaphase or telophase (Fig. 3). These results suggest that depletion of SGO1 increases mitotic arrest, or slows down the transition from metaphase to anaphase, and from anaphase to telophase. These results are expected given the described functions of SGO1 in the regulation of chromatid cohesion.

### The downregulation of SGO1 suppresses EMT, cell invasion, and migration

**Fig. 4:**
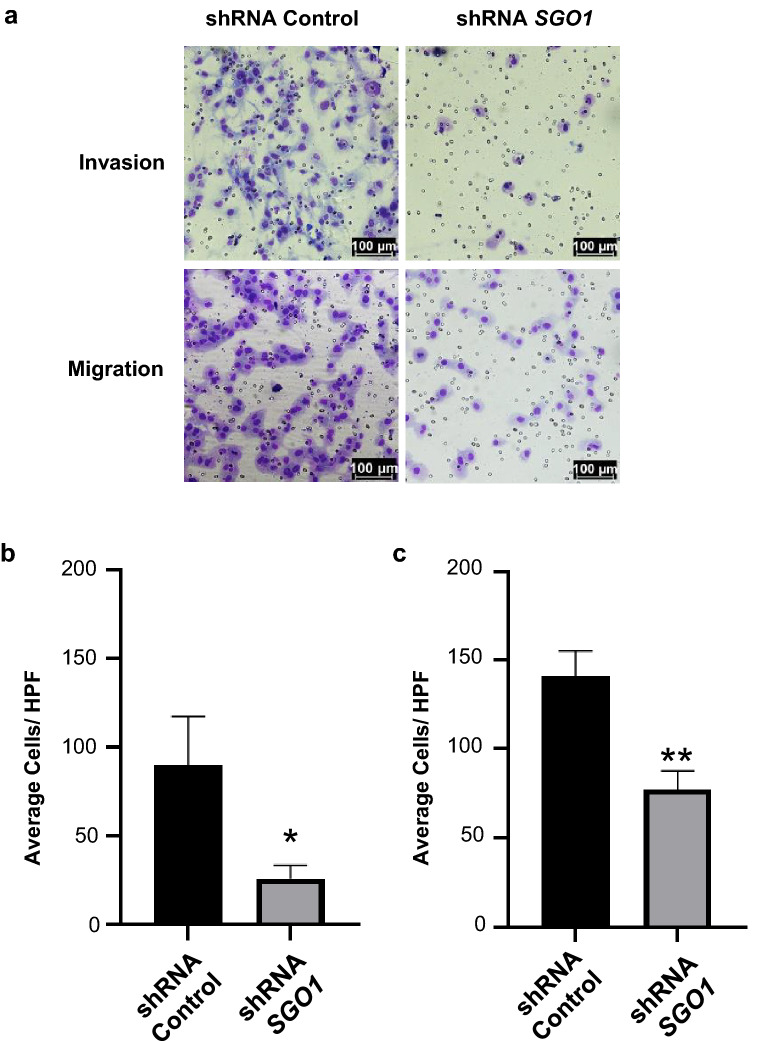
SGO1 promotes cell invasion and migration in triple-negative breast cancer cells. Representative images of invading (top) or migrating (bottom) shRNA Control or shRNA *SGO1* MDA-MB-231 cells (**a**). Images were taken at 200X magnification (scale bars = 100 μm). Graphs represent the average of triplicates of cells that invaded (**b**) and migrated **(c)**. The error bars represent the mean ± SEM. *p < 0.05, **p < 0.01, and ***p < 0.001 were considered statistically significant. N.S. stands for not significant. A Student t-test (unpaired, two-tailed) was used to compare differences between groups

To investigate the mechanisms by which the downregulation of SGO1 suppresses metastasis, we investigated the effects of SGO1 downregulation on cell invasion and cell migration. Results from Fig. 4a–c demonstrate that SGO1 downregulation significantly reduces cell invasion and migration.

**Fig. 5:**
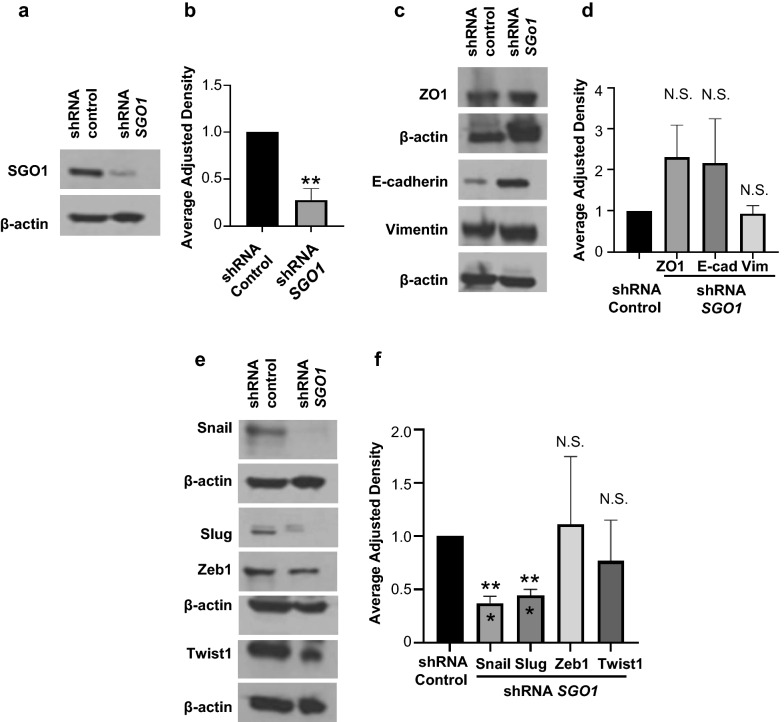
SGO1 modulates epithelial-to-mesenchymal protein expression. Western blot showing characterization of shRNA *SGO1* in MDA-MB-231 cells (**a**). The graph shows the densitometry of the Western blot analysis (**b**). Western blot showing the expression of epithelial markers E-cadherin (E-cad) and ZO1, and mesenchymal marker Vimentin (Vim) in MDA-MB-231 cells treated with shRNA Control or shRNA *SGO1* (**c**). The graph shows the densitometry of the Western blot analysis (**d**). Western blot showing the expression of EMT transcription factors Snail, Slug, Zeb1, and Twist1 in MDA-MB-231 cells treated with shRNA Control or shRNA SGO1 (**e**). The graph shows the densitometry of the Western blot analysis (**f**). For all graphs, error bars represent mean ± SEM. *p < 0.05, **p < 0.01, and ***p < 0.001 were considered statistically significant. N.S. stands for not significant. A Student t-test (unpaired, two-tailed) was used to compare differences between groups

We hypothesized that these changes were mediated through EMT protein modulation, and through transcription factors that promote EMT. Therefore, we evaluated how SGO1 downregulation (Figs. 5a, b) affected the expression of certain EMT-related proteins including the epithelial markers E-cadherin and ZO1, and the mesenchymal marker Vimentin. We observed an increase in epithelial markers E-cadherin and ZO1, but it was not statistically significant. Meanwhile, no changes were observed for Vimentin (Figs. 5c, d). On the other hand, we did observe that SGO1 downregulation significantly decreases Snail and Slug protein expression, while the changes in other transcription factors that promote EMT (Zeb1 and Twist) were unchanged (Figs. 5e, f).

**Fig. 6:**
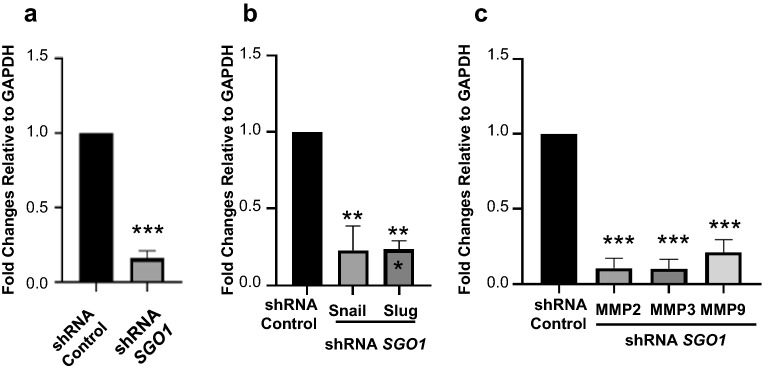
SGO1 modulates EMT mRNA expression. Graphs show the average mRNA fold changes relative to GAPDH (obtained by quantitative real-time PCR) of *SGO1* (**a**), EMT transcription factors (*Snail* and Slug) (**b**), and MMP (*MMP2*, *MMP3*, and *MMP9*) (**c**) mRNA expression in shRNA Control or shRNA *SGO1* MDA-MB-231 cells. Experiments were done as biological triplicates. Error bars represent mean ± SEM. *p < 0.05, **p < 0.01, and ***p < 0.001 were considered statistically significant. N.S. stands for not significant. A Student t-test (unpaired, two-tailed) was used to compare differences between groups

Moreover, SGO1 downregulation (Fig. 6a) leads to a significant reduction in mRNA levels of the EMT transcription factors Snail and Slug (Fig. 6b). Furthermore, SGO1 downregulation significantly reduces the mRNA levels of MMP2, MMP3, and MMP9 (Fig. 6c). These MMPs are also important mediators of cell invasion and migration through the degradation of the extracellular matrix (ECM). Because our laboratory has previously shown that the downregulation of Snail, Slug, and MMP3 significantly suppress cell invasion and migration in TNBC cells [25, 27], these results suggest that SGO1 downregulation reduces cell invasion and migration through the downregulation of EMT transcription factors belonging to the Snail family (Snail and Slug) and MMPs (MMP2, MMP3, and MMP9).

### The downregulation of SGO1 decreases cell proliferation and viability in TNBC

**Fig. 7:**
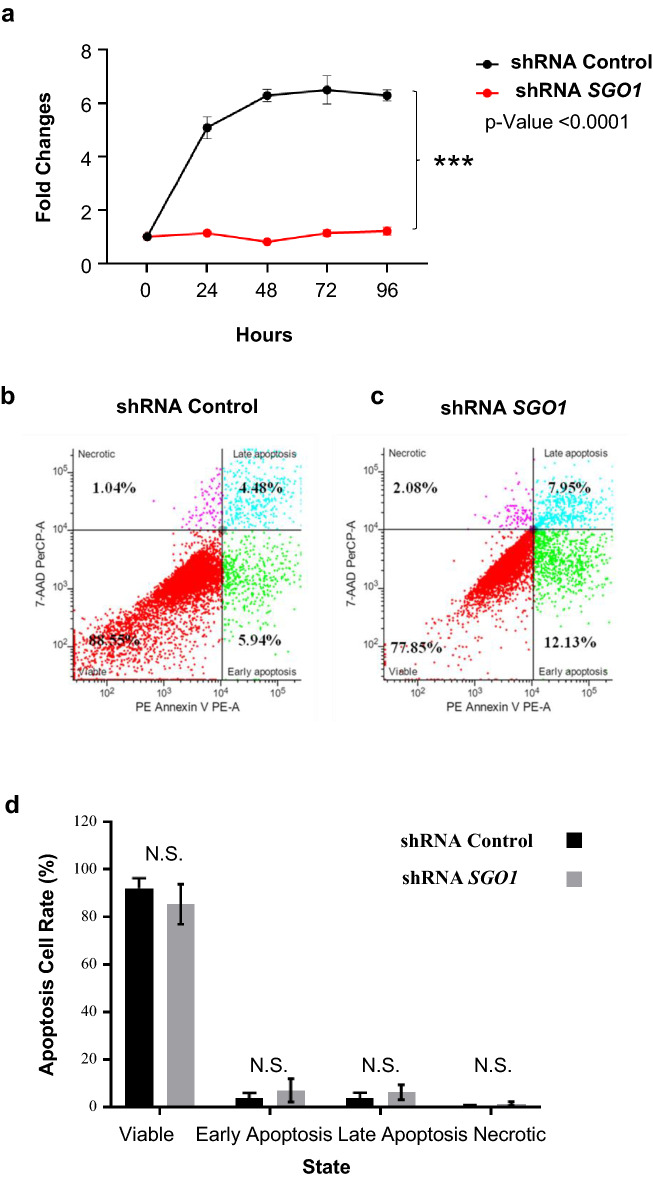
SGO1 downregulation significantly decreases cell proliferation and viability. Graph showing the average cell proliferation and viability for MDA-MB-231 cells treated with shRNA *SGO1* compared to shRNA Control (**a**). Error bars represent mean ± SEM. *p < 0.05, **p < 0.01, and ***p < 0.001 were considered statistically significant. A Student t-test (unpaired, two-tailed) was used to compare differences between groups. Cell sorting analysis showing the percentage of viable, necrotic, early apoptotic, or late apoptotic shRNA Control (**b**) or shRNA *SGO1* (**c**) MDA-MB-231 cells. Graph showing the average of three experiments (**d**). Error bars represent mean ± SEM. *p < 0.05, **p < 0.01, and ***p < 0.001 were considered statistically significant. N.S. stands for not significant. A Student t-test (unpaired, two-tailed) was used to compare differences between groups

Lastly, we explored the effects of SGO1 downregulation on cell proliferation, cell viability, and apoptosis. Given its role in cell division and the marked effect on tumor growth, we hypothesized that the downregulation of SGO1 will affect cell viability. Figure 7a shows that the downregulation of SGO1 significantly reduces cell proliferation and viability. However, differences between control and shRNA *SGO1* in cells undergoing early and late apoptosis as well as in necrosis were not significant (Fig. 7b–d). Taken together, these results suggest that SGO1 downregulation has a role in cell proliferation and viability but not in apoptosis. Nevertheless, the results suggest that targeting SGO1 may be helpful for early disease treatment by limiting cell proliferation and viability.

## Discussion

Metastasis in TNBC remains a clinical challenge due to the lack of effective therapies. Previous studies from our laboratory showed roles for SGO1 in centrosome amplification, cell invasion, cell migration, and EMT [24, 26]. However, this is the first study that shows a role for SGO1 in tumor growth and metastasis in TNBC.

Our group pioneered the study of SGO1 in breast cancer in general by demonstrating, using cBIOPORTAL, that the overexpression of SGO1 in breast tumors correlates with the overexpression of the E2F transcriptional activators E2F1, E2F2, and E2F3 [23]. The E2F activator overexpression is higher in the basal/TNBC subtype, which may contribute to the highly proliferative nature of these tumors. The study from Lee et al. [23] also demonstrated that E2F1 and E2F3 overexpression in MCF10A (non-transformed human mammary epithelial cells) correlated with increased expression of SGO1. Because the role of SGO1 in TNBC was unknown, Jusino et al. [26], demonstrated, using cBIOPORTAL,  that SGO1 is more frequently overexpressed in patients with basal/TNBC relative to other subtypes (30% of basal/TNBC patients vs. 0.5% in Luminal A and 6.3% of Luminal B patients); this follows very closely the pattern of expression of E2F3 in TNBC (42% in basal/TNBC, 0.5% in Luminal A and 2.42% Luminal B). Therefore, we compared E2F3 and one of its targets, SGO1, in their ability to suppress early metastasis (EMT, cell invasion, and cell migration) and demonstrated that their transient downregulation suppressed early metastasis.

In the present manuscript, we demonstrate that the sustained downregulation of SGO1 can suppress metastasis. We were encouraged by the results published by our laboratory that the sustained downregulation of E2F3 slowed down the growth of TNBC tumors significantly, but slightly over controls [25]. What may allow the growth of cells with downregulated E2F3 is the presence of the other E2F activators, E2F2 and E2F1; in other words, the functional redundancy among the E2F activators. In contrast, SGO1 is a mitotic protein with a unique function -holding sister chromatids together [30]. Therefore, the stable silencing of SGO1 greatly reduces tumor growth, while suppressing metastasis. Future experiments can be done by allowing tumors to grow and inducibly knocking down or knocking out SGO1.

Our results suggest that the suppression of tumor growth and metastasis in TNBC following SGO1 downregulation are in part mediated by Snail and Slug. In other publications, we demonstrated the direct roles of these transcription factors in mediating early metastasis in TNBC [25, 27]. The Snail family (Snail and Slug) functions as master regulators of EMT by repressing several epithelial genes including E-cadherin while activating mesenchymal genes such as N-cadherin. The Snail family also activates MMPs and other transcription factor families (e.g., Twist and Zeb) (reviewed in [31]). Further, we demonstrated the key role of SGO1 in the regulation of MMP2, MMP3, and MMP9, which are important to modulate cell invasion and migration. In a previous publication, our lab demonstrated that MMP3 is critical to driving cell migration and invasion downstream of SGO1 in TNBC [25]. Moreover, we demonstrated the important role of SGO1 in cell proliferation and viability. However, despite observing some increase in apoptosis and necrosis, these changes were not significant. This suggests that SGO1 affects cell viability through proliferation. Another explanation is that SGO1 may reduce tumor growth (and thus metastasis) due to its role in cell division. This is suggested by experiments presented in Fig. 3b that in vivo tumors that are downregulated for SGO1 have an increased number of cells in metaphase and anaphase relative to shRNA controls; this suggests either cell cycle arrest or a change in mitotic timing. Future studies can be performed to further elucidate this cross-talking mechanism that links the cell cycle and metastasis.

Our study shows a promising link between SGO1, EMT, and metastasis in TNBC. Thus, suggesting that SGO1 may serve as a therapeutic target for metastatic TNBC. Alternatively, proteins that interact with SGO1 (e.g., Aurora B, Bub1, Nek2, or TTK) [32] can be targeted with small molecule inhibitors. Targeting any of these kinases may be more feasible than targeting SGO1, which is not a kinase, and already some of these kinases are in clinical trials (reviewed in [33]). Moreover, we have shown the role of Nek2 [27] and TTK [28] in EMT. Therefore, the biological inactivation of SGO1, or of kinases that can interact with SGO1 or that phosphorylate SGO1, can be used as a future therapeutic strategy against TNBC.

## Conclusion

**Fig. 8:**
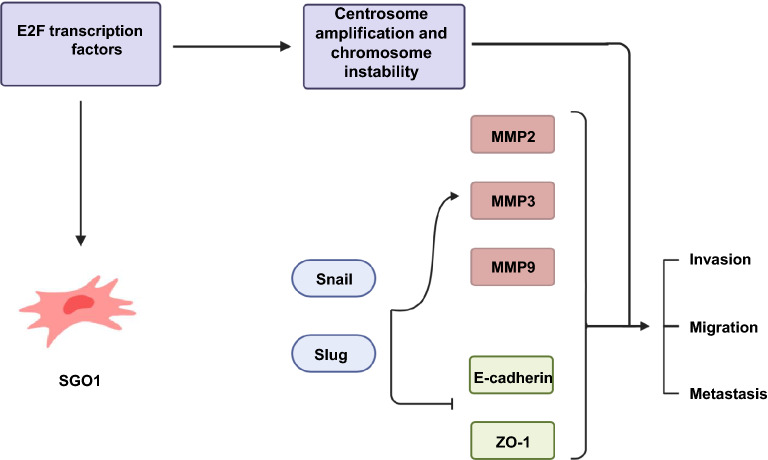
Research model. Diagram summarizing our findings that SGO1 modulates Snail and Slug expression, which in turn leads to EMT through the repression of epithelial markers and activates mesenchymal markers, including MMPs. In turn, these molecular changes act as precursors to cell invasion, cell migration, and metastasis in TNBC

Our findings indicate that SGO1 plays a novel role in TNBC by regulating the transcription factors Snail and Slug. These transcription factors in turn modulate EMT genes (including MMP2, MMP3, and MMP9), cell invasion, migration, and lung metastasis (Fig. 8). Therefore, SGO1 and upstream regulators of SGO1 represent an opportunity to develop targeted therapies for TNBC.

## Supplementary Information

**Additional file 1: Figure S1 a-b.** Representative images of primary tumors recovered from NSG mice treated with MDA-MB-231 cells expressing shRNA Control (5-10) or shRNA *SGO1* (15-20). **Figure S2.** Original Western blot films showing SGO1 **(a)** and β-actin **(b)** expression in tumor lysates from NSG mice treated with MDA-MB-231 cells expressing shRNA Control (1-4) or shRNA *SGO1* (5-8). **Figure S3.** Original Western blot films showing SGO1 **(a)**, Snail **(b)**, and β-actin **(c)** expression in MDA-MB-231 cells expressing shRNA Control (1, 3, and 5) or shRNA *SGO1* (2, 4, and 6) from three independent experiments. **Figure S4.** Original Western blot films showing Slug **(a)**, Zeb1 **(b)**, and β-actin **(c) **expression in MDA-MB-231 cells expressing shRNA Control (1, 3, and 5) or shRNA *SGO1* (2, 4, and 6) from three independent experiments. **Figure S5.** Original Western blot films showing Tiwst1 **(a)** and β-actin **(b) **expression in MDA-MB-231 cells expressing shRNA Control (1, 3, and 5) or shRNA *SGO1* (2, 4, and 6) from three independent experiments. **Figure S6.** Original Western blot films showing ZO1 **(a)** and β-actin **(b)** expression in MDA-MB-231 cells expressing shRNA Control (1, 3, and 5) or shRNA SGO1 (2, 4, and 6) from three independent experiments. **Figure S7.** Original Western blot films showing E-cadherin and Vimentin **(a)** and β-actin **(b)** expression in MDA-MB-231 cells expressing shRNA Control (1, 3, and 5) or shRNA SGO1 (2, 4, and 6) from three independent experiments.

## Data Availability

All the data generated as part of this study are included in this published article and its Additional files.
