# Supplementary material for: Sustained Shugoshin 1 downregulation reduces tumor growth and metastasis in a mouse xenograft tumor model of triple-negative breast cancer
Source: Cell Div. 2023 Apr 30;18:6. doi: 10.1186/s13008-023-00088-5 (PMC10150544; doi:10.1186/s13008-023-00088-5)
Supplement: Supplementary file 1 — Additional file 1: Figure S1 a-b. Representative images of primary tumors recovered from NSG mice treated with MDA-MB-231 cells expressing shRNA Control (5-10) or shRNA SGO1 (15-20). Figure S2. Original Western blot films showing SGO1 (a) and β-actin (b) expression in tumor lysates from NSG mice treated with MDA-MB-231 cells expressing shRNA Control (1-4) or shRNA SGO1 (5-8). Figure S3. Original Western blot films showing SGO1 (a), Snail (b), and β-actin (c) expression in MDA-MB-231 cells expressing shRNA Control (1, 3, and 5) or shRNA SGO1 (2, 4, and 6) from three independent experiments. Figure S4. Original Western blot films showing Slug (a), Zeb1 (b), and β-actin (c) expression in MDA-MB-231 cells expressing shRNA Control (1, 3, and 5) or shRNA SGO1 (2, 4, and 6) from three independent experiments. Figure S5. Original Western blot films showing Tiwst1 (a) and β-actin (b) expression in MDA-MB-231 cells expressing shRNA Control (1, 3, and 5) or shRNA SGO1 (2, 4, and 6) from three independent experiments. Figure S6. Original Western blot films showing ZO1 (a) and β-actin (b) expression in MDA-MB-231 cells expressing shRNA Control (1, 3, and 5) or shRNA SGO1 (2, 4, and 6) from three independent experiments. Figure S7. Original Western blot films showing E-cadherin and Vimentin (a) and β-actin (b) expression in MDA-MB-231 cells expressing shRNA Control (1, 3, and 5) or shRNA SGO1 (2, 4, and 6) from three independent experiments. [file 13008_2023_88_MOESM1_ESM.pptx]

## Slide 1
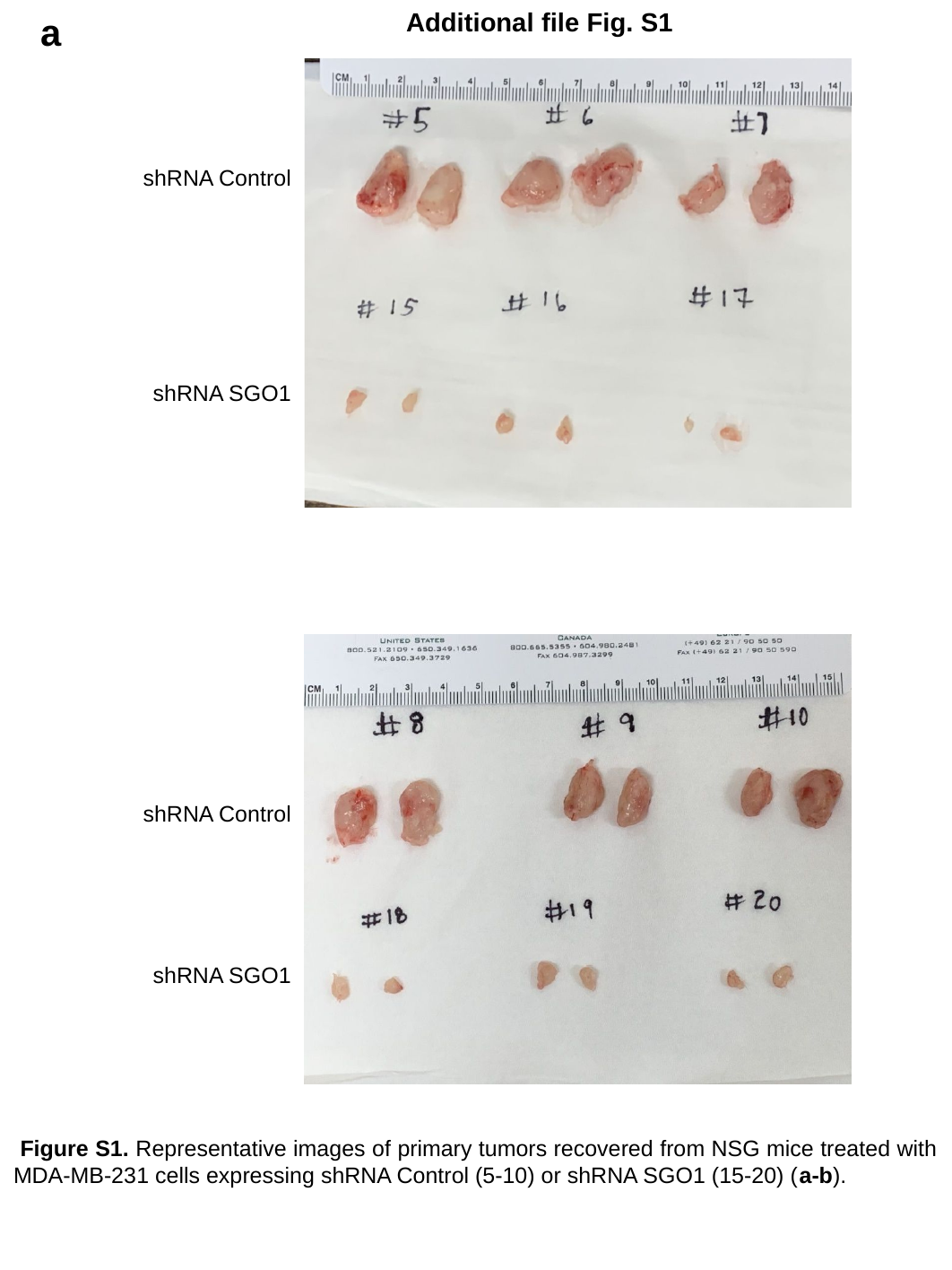

Additional file Fig. S1
a
 b
shRNA Control
shRNA SGO1
shRNA Control
shRNA SGO1
 Figure S1. Representative images of primary tumors recovered from NSG mice treated with MDA-MB-231 cells expressing shRNA Control (5-10) or shRNA SGO1 (15-20) (a-b).

## Slide 2
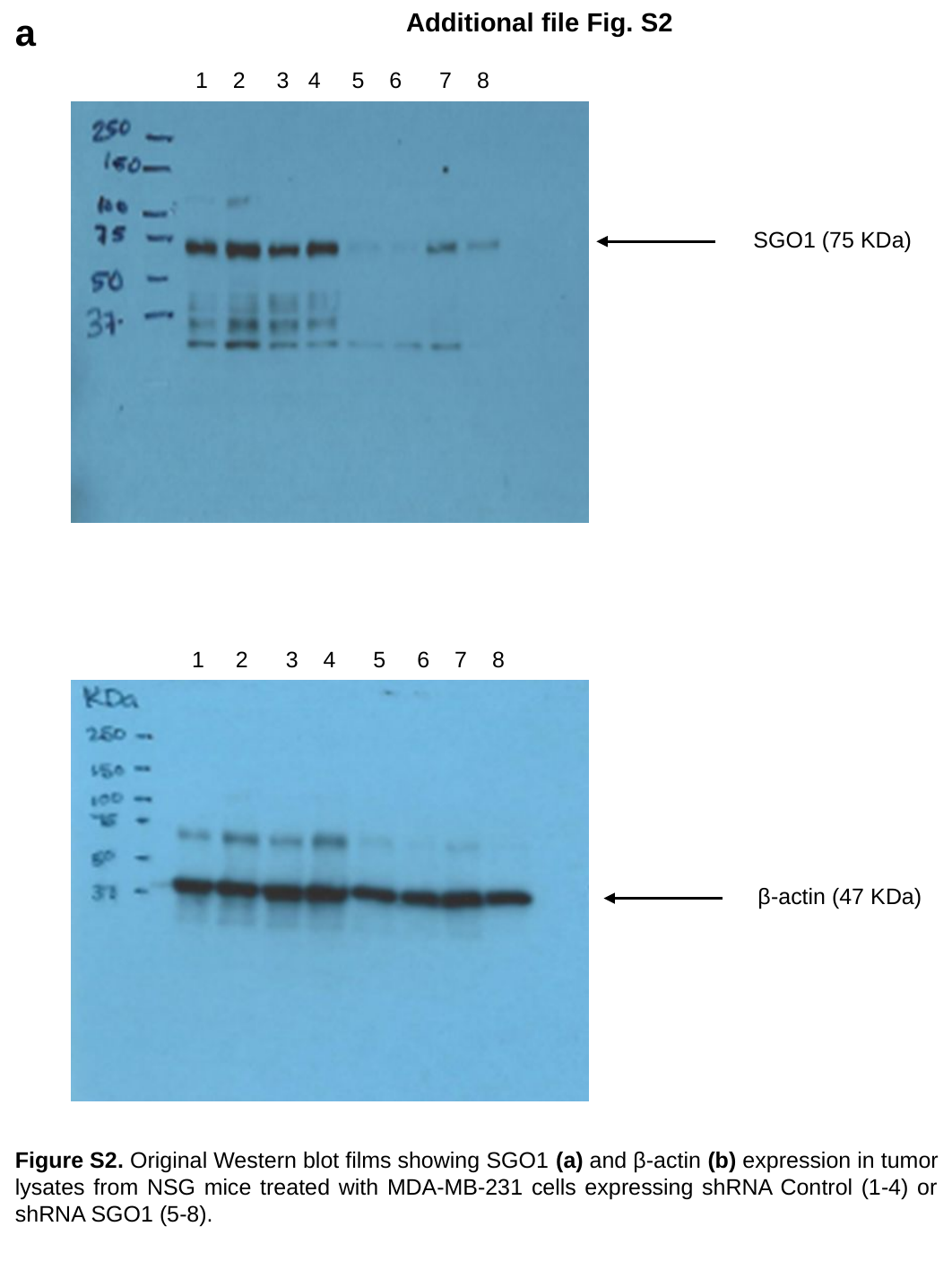

Additional file Fig. S2
a
 b
 1 2 3 4 5 6 7 8
SGO1 (75 KDa)
 1 2 3 4 5 6 7 8
β-actin (47 KDa)
Figure S2. Original Western blot films showing SGO1 (a) and β-actin (b) expression in tumor lysates from NSG mice treated with MDA-MB-231 cells expressing shRNA Control (1-4) or shRNA SGO1 (5-8).

## Slide 3
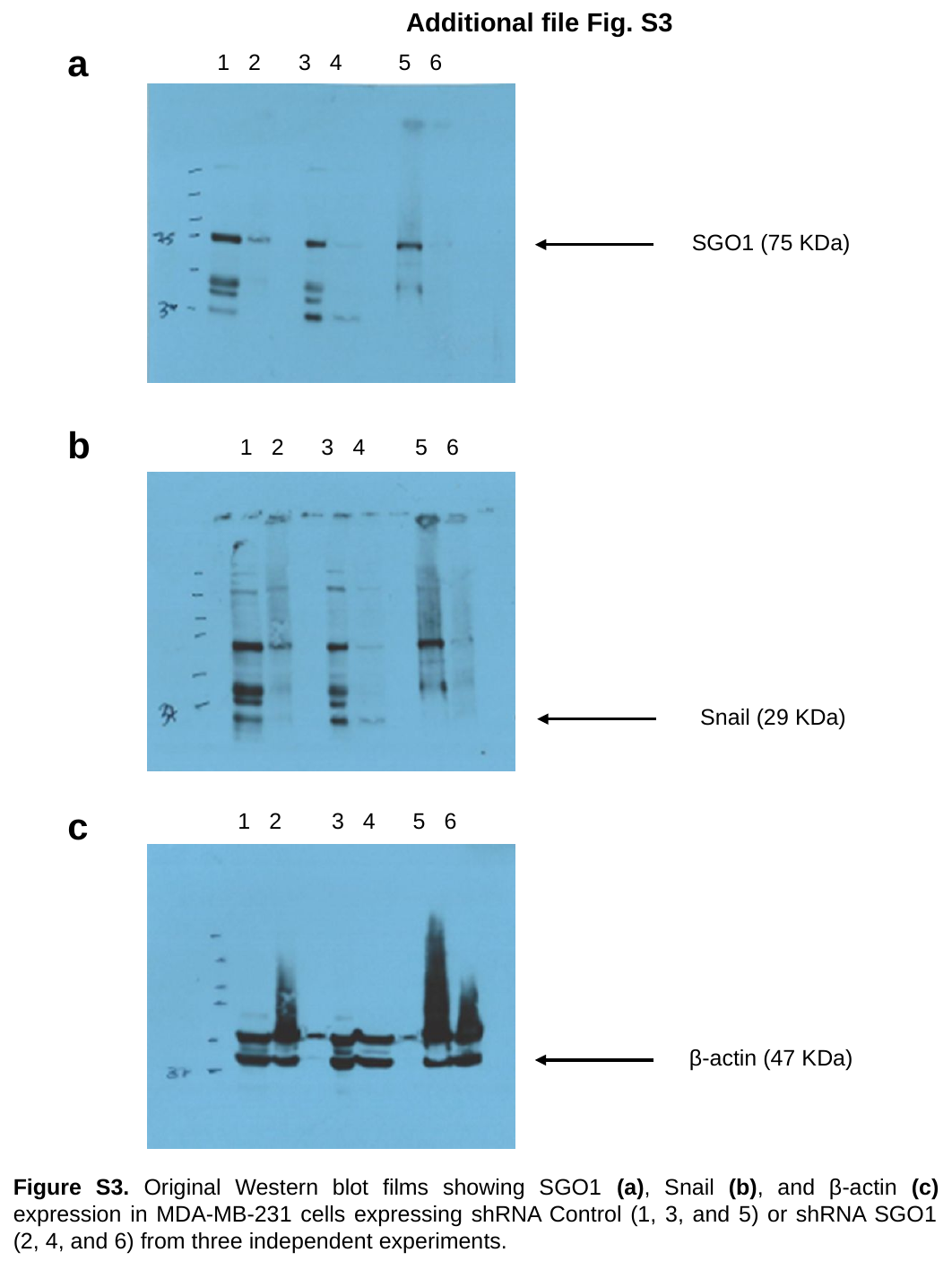

Additional file Fig. S3
a
b
c
 1 2 3 4 5 6
SGO1 (75 KDa)
1 2 3 4 5 6
Snail (29 KDa)
1 2 3 4 5 6
β-actin (47 KDa)
Figure S3. Original Western blot films showing SGO1 (a), Snail (b), and β-actin (c) expression in MDA-MB-231 cells expressing shRNA Control (1, 3, and 5) or shRNA SGO1 (2, 4, and 6) from three independent experiments.

## Slide 4
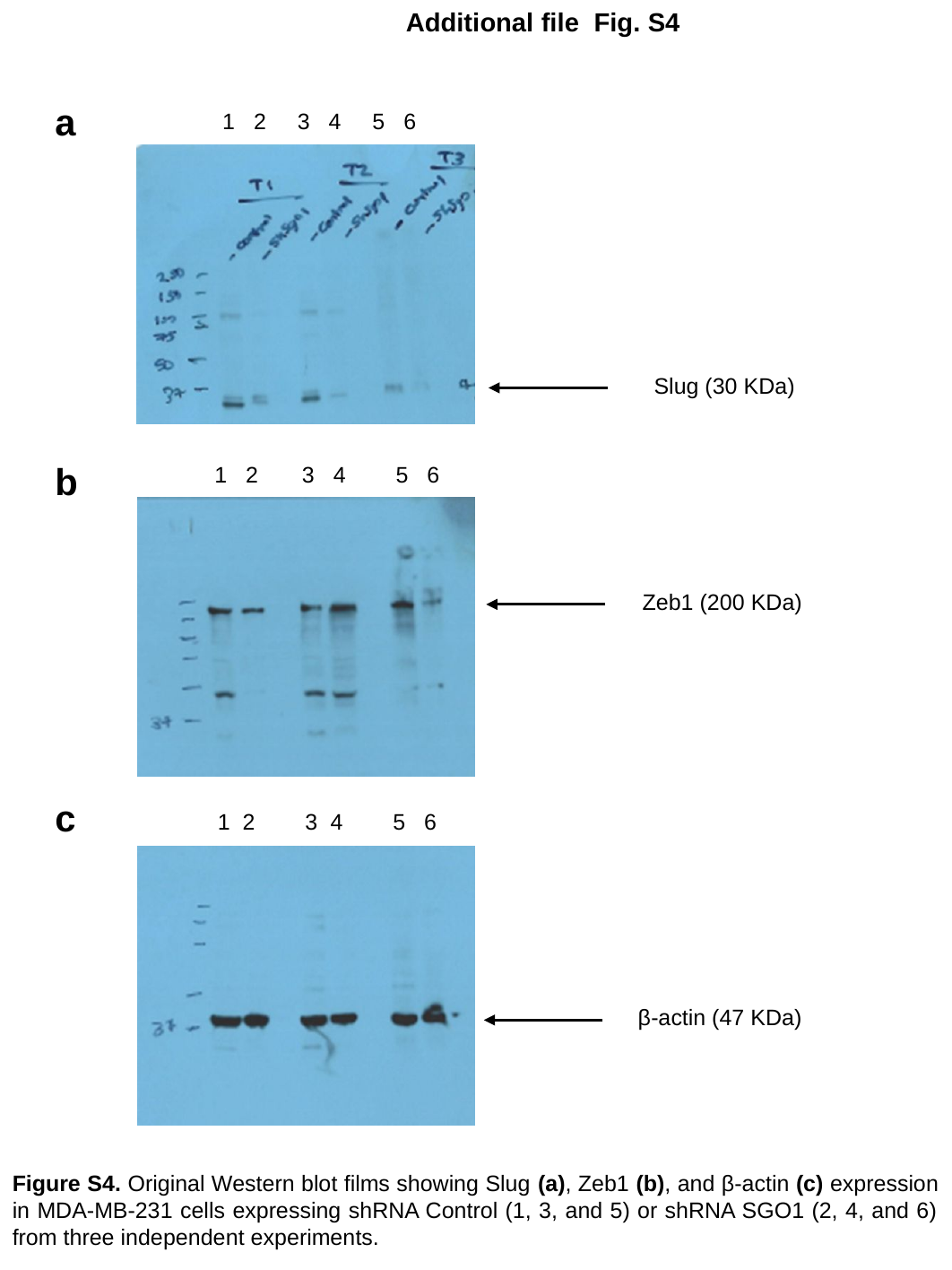

Additional file Fig. S4
a
b
c
1 2 3 4 5 6
Slug (30 KDa)
1 2 3 4 5 6
Zeb1 (200 KDa)
1 2 3 4 5 6
β-actin (47 KDa)
Figure S4. Original Western blot films showing Slug (a), Zeb1 (b), and β-actin (c) expression in MDA-MB-231 cells expressing shRNA Control (1, 3, and 5) or shRNA SGO1 (2, 4, and 6) from three independent experiments.

## Slide 5
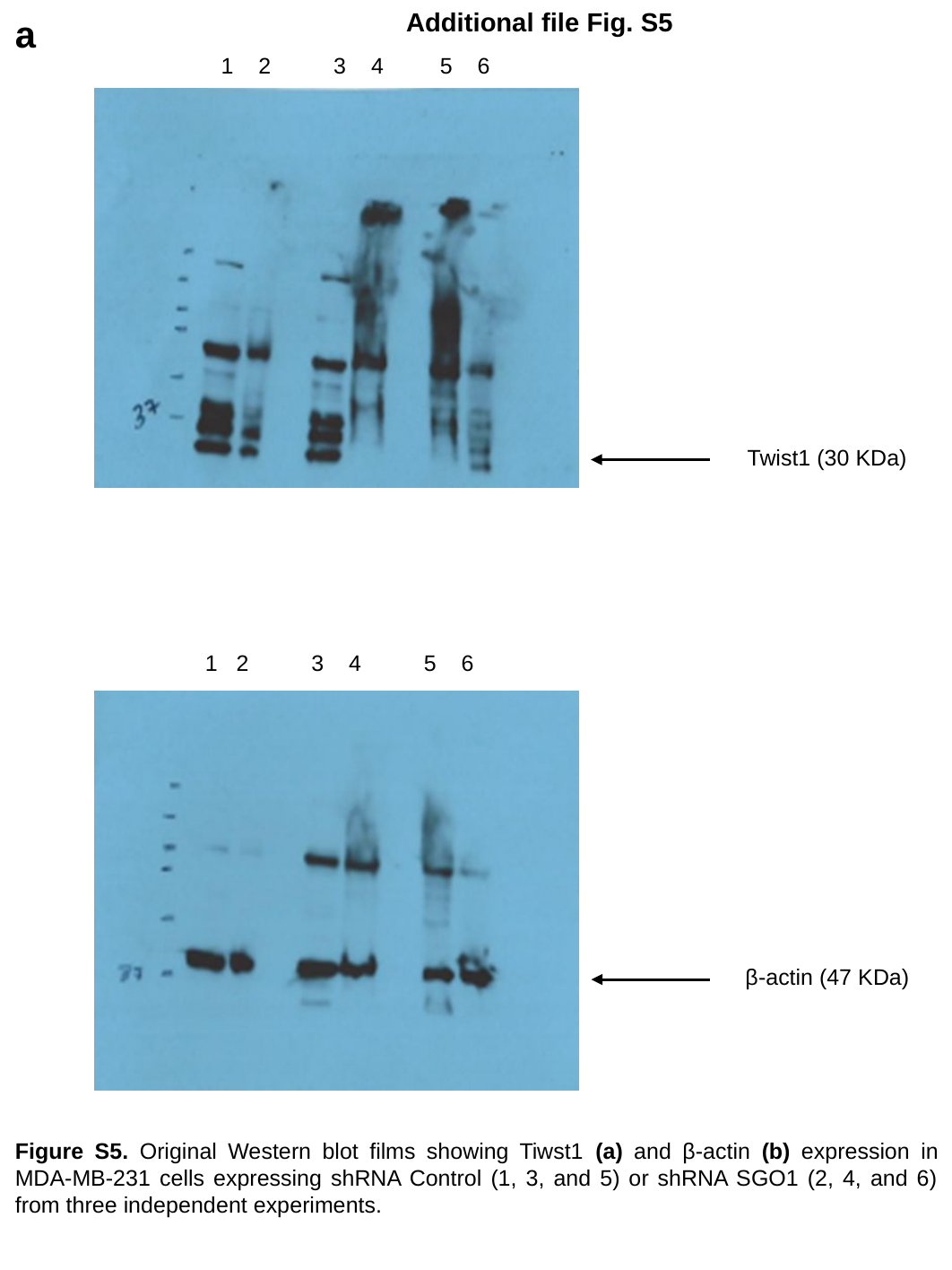

Additional file Fig. S5
a
 b
 1 2 3 4 5 6
Twist1 (30 KDa)
1 2 3 4 5 6
β-actin (47 KDa)
Figure S5. Original Western blot films showing Tiwst1 (a) and β-actin (b) expression in MDA-MB-231 cells expressing shRNA Control (1, 3, and 5) or shRNA SGO1 (2, 4, and 6) from three independent experiments.

## Slide 6
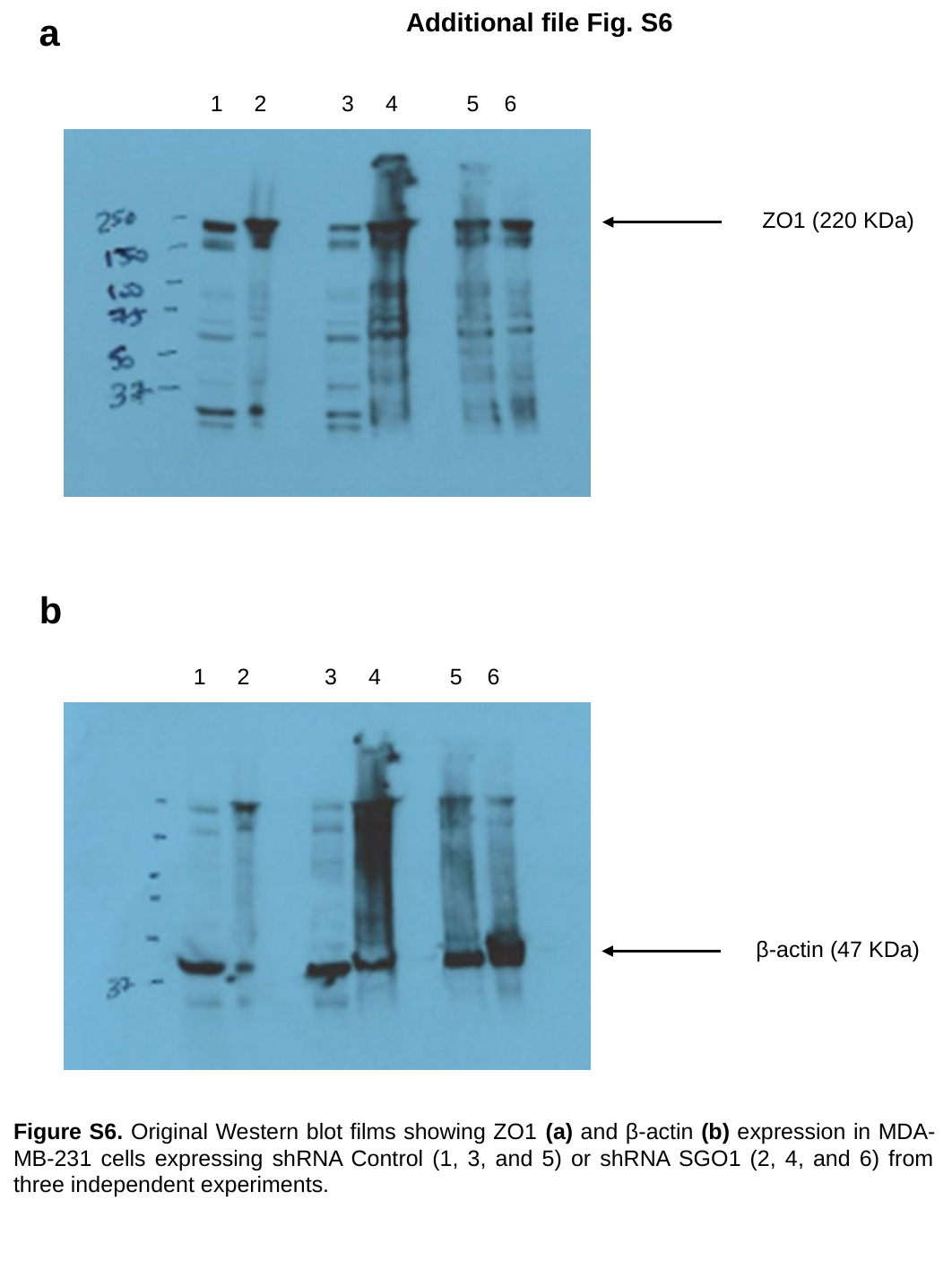

Additional file Fig. S6
a
1 2 3 4 5 6
ZO1 (220 KDa)
b
1 2 3 4 5 6
β-actin (47 KDa)
Figure S6. Original Western blot films showing ZO1 (a) and β-actin (b) expression in MDA-MB-231 cells expressing shRNA Control (1, 3, and 5) or shRNA SGO1 (2, 4, and 6) from three independent experiments.

## Slide 7
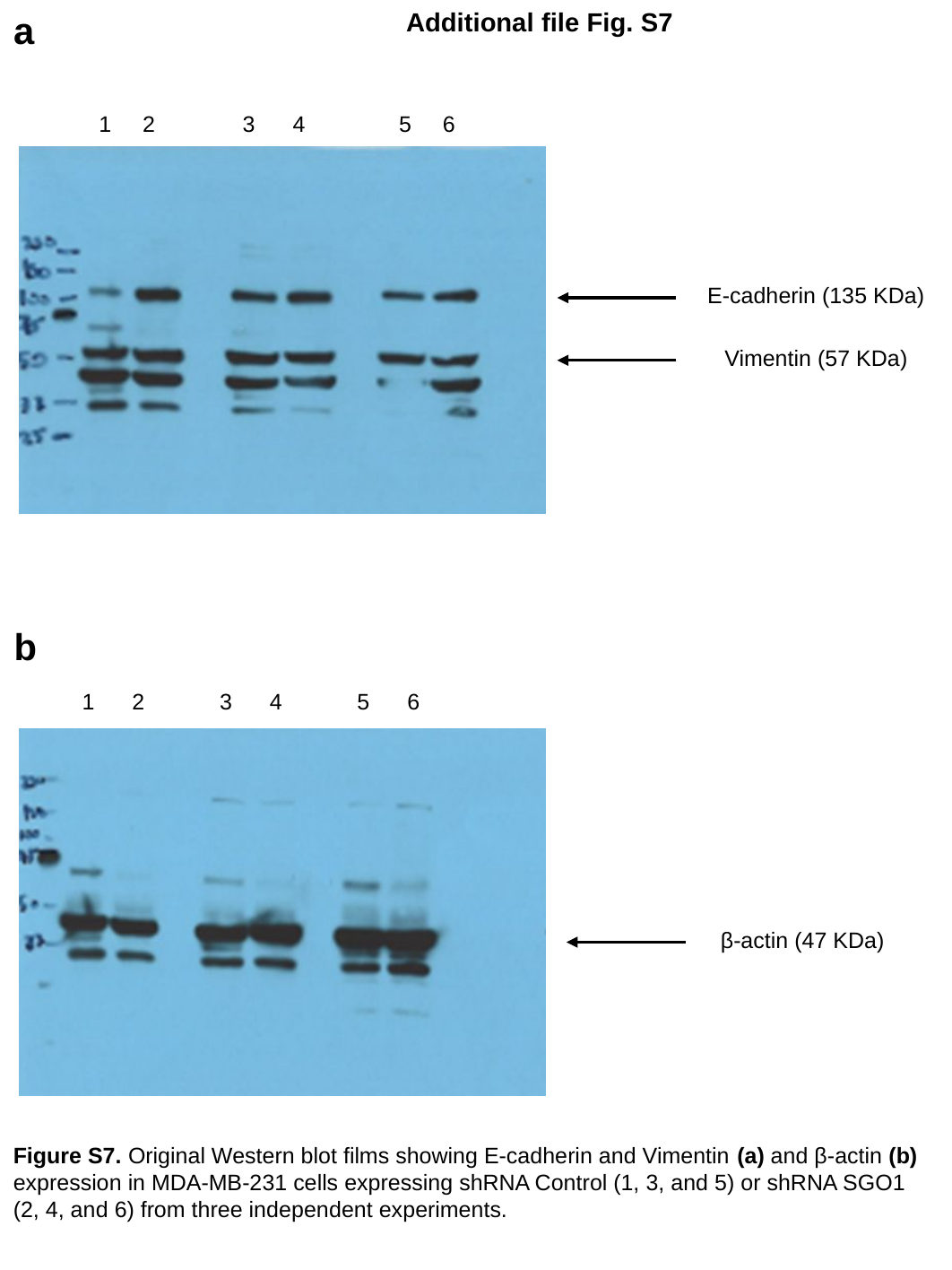

Additional file Fig. S7
a
 1 2 3 4 5 6
E-cadherin (135 KDa)
Vimentin (57 KDa)
b
 1 2 3 4 5 6
β-actin (47 KDa)
Figure S7. Original Western blot films showing E-cadherin and Vimentin (a) and β-actin (b) expression in MDA-MB-231 cells expressing shRNA Control (1, 3, and 5) or shRNA SGO1 (2, 4, and 6) from three independent experiments.
